# Intra- and extra-cellular environments contribute to the fate of HIV-1 infection

**DOI:** 10.1016/j.celrep.2021.109622

**Published:** 2021-08-31

**Authors:** Sneha Ratnapriya, Miranda Harris, Angela Chov, Zachary T. Herbert, Vladimir Vrbanac, Maud Deruaz, Vasudevan Achuthan, Alan N. Engelman, Joseph Sodroski, Alon Herschhorn

**Affiliations:** 1 Division of Infectious Diseases and International Medicine, Department of Medicine, University of Minnesota, Minneapolis, MN 55455, USA; 2 Molecular Biology Core Facilities, Dana-Farber Cancer Institute, Boston, MA 02215, USA; 3 Humanized Immune System Mouse Program, Ragon Institute of MGH, MIT and Harvard, and Center for Immunology and Inflammatory Disease, Cambridge, MA 02139, USA; 4 Department of Cancer Immunology and Virology, Dana-Farber Cancer Institute, Boston, MA 02215, USA; 5 Department of Medicine, Harvard Medical School, Boston, MA 02115, USA; 6 Department of Microbiology, Harvard Medical School, Boston, MA 02115, USA; 7 Institute for Molecular Virology, University of Minnesota, Minneapolis, MN 55455, USA; 8 Lead contact

## Abstract

HIV-1 entry into host cells leads to one of the following three alternative fates: (1) HIV-1 elimination by restriction factors, (2) establishment of HIV-1 latency, or (3) active viral replication in target cells. Here, we report the development of an improved system for monitoring HIV-1 fate at single-cell and population levels and show the diverse applications of this system to study specific aspects of HIV-1 fate in different cell types and under different environments. An analysis of the transcriptome of infected, primary CD4+ T cells that support alternative fates of HIV-1 identifies differential gene expression signatures in these cells. Small molecules are able to selectively target cells that support viral replication with no significant effect on viral latency. In addition, HIV-1 fate varies in different tissues following infection of humanized mice *in vivo*. Altogether, these studies indicate that intra- and extra-cellular environments contribute to the fate of HIV-1 infection.

## In brief

Ratnapriya et al. provide evidence that complex interactions of HIV-1 with the host in target cells and within the cellular environment contribute to HIV-1 fate and can be selectively modulated by small molecules.

## Graphical Abstract

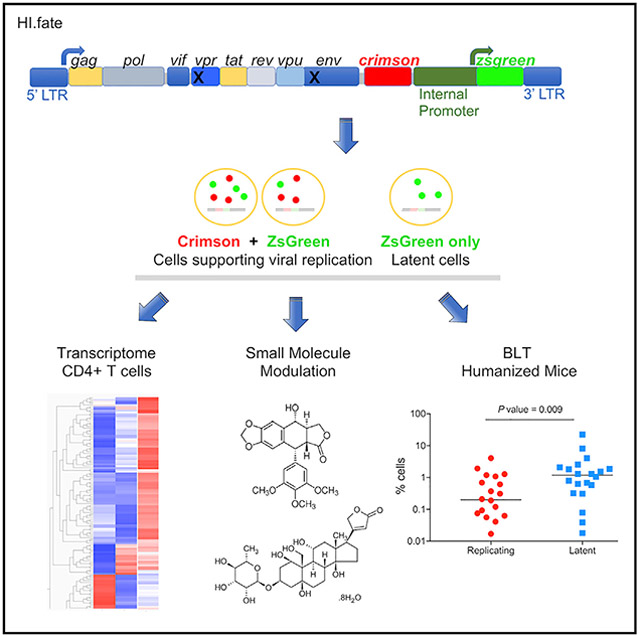

## INTRODUCTION

**Figure 1.**
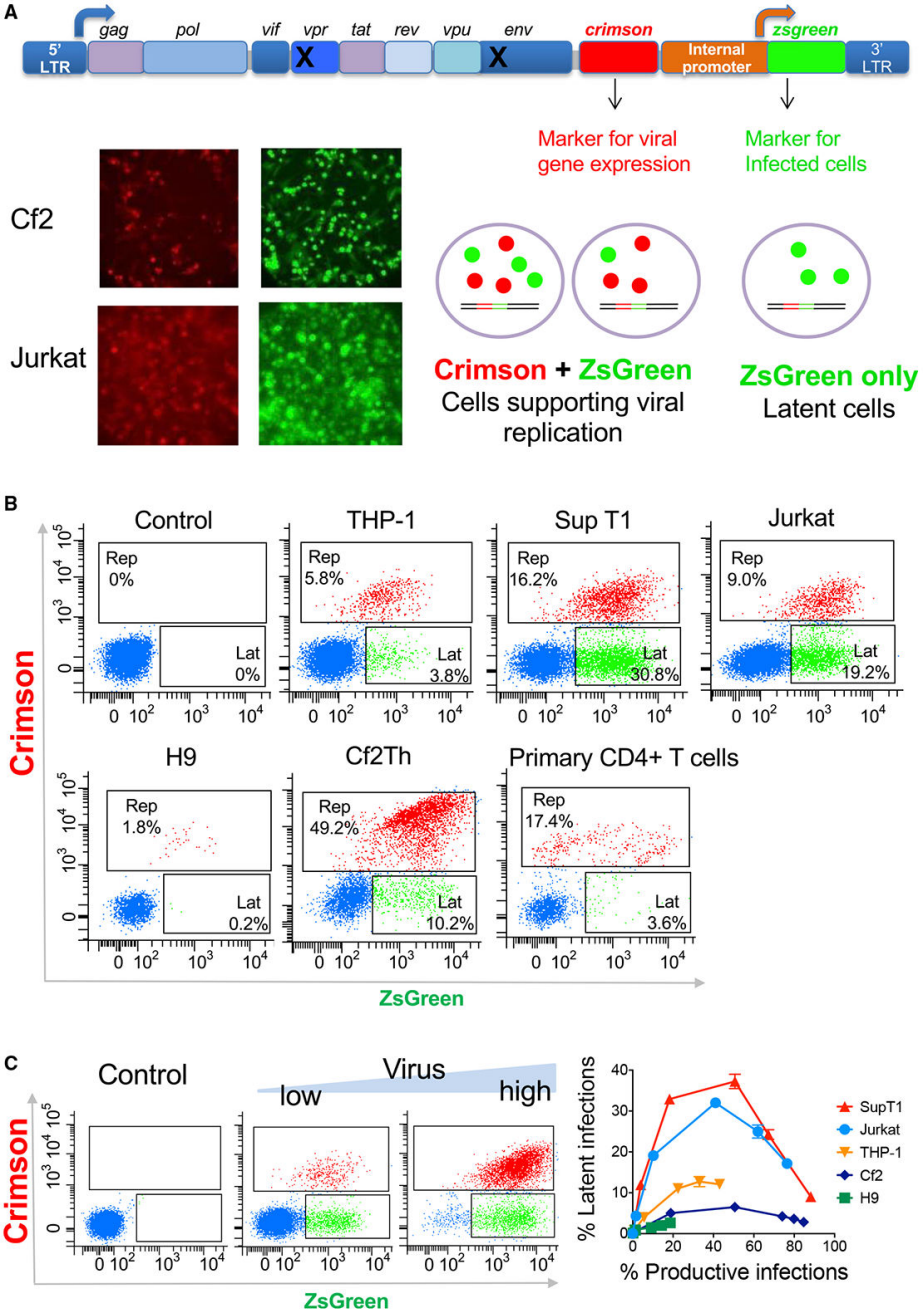
An improved HIV-1 reporter vector (HI.fate) distinguishes alternative fates of HIV-1 infection in target cells (A) HI.fate is based on the chimeric molecular clone HIV-1_NL4-3_, which contains the 5′-long terminal repeat (LTR) from the NY5 isolate and the 3′-LTR from the LAV isolate. Differences between the two LTR sequences decrease the frequency of undesirable recombination events during vector manipulations. Bottom: infection of different cells by HI.fate.E (EF1a-HTLV chimeric internal promoter) viruses leads to the expression of E2-Crimson and ZsGreen fluorescent proteins, which are visualized under a fluorescence microscope. (B) Flow cytometric analysis of different cells infected by HI.fate.E viruses. THP-1 cells were differentiated using 100 nM phorbol myristate acetate (PMA); primary CD4 T cells were isolated from peripheral blood mononuclear cells (PBMCs) by negative selection and activated with anti-CD3/CD28 beads prior to infection. Cells that support HIV-1 gene expression are shown in red and those supporting HIV-1 latency are shown in green. Uninfected cells are colored blue. (C) Increasing amounts of HI.fate.E viruses were used to infect target cells. Left panel shows an example of the readout for 10,000 cells. In the right panel, fluorescence profile of virus titers was plotted for each cell type (mean ± SD of percentage of cells exhibiting a speficied phenotype in a representative experiment). Results represent one of two independent experiments, each performed in duplicate, except for infection of THP-1 cells that were tested in a single experiment performed in duplicate.

Cells containing a latent HIV-1 provirus are rare in people living with HIV (PLWH), and only a small fraction of these cells carry a replication-competent provirus capable of replenishing the viral population upon HIV-1 reactivation (Chun et al., 1997; Wong et al., 1997). Very low number of latent cells makes it extremely difficult to identify and isolate these cells from samples of PLWH (Bruner et al., 2019; Ho et al., 2013). Thus, several model systems have been developed in recent years to investigate and provide insights into the establishment and maintenance of the latent HIV-1 reservoir (Darcis et al., 2015; Spina et al., 2013). A recent and popular system uses a reporter HIV-1-based vector in which one fluorescent protein is transcribed from the HIV-1 long terminal repeat (LTR) promoter to monitor HIV-1-mediated transcription and a second fluorescent protein is transcribed from an internal promoter to report HIV-1 entry and provirus integration in infected cells (Calvanese et al., 2013; Chavez et al., 2015). Here, we used similar building blocks to construct a system, designated HI.fate, with improved elements to simultaneously monitor HIV-1 replication and latency at single-cell and population levels (Figure 1A; Ratnapriya et al., 2020a; Ukah et al., 2018). Our reporter system is based on the NL4-3 backbone and maintains genetic stability as a result of differences between the upstream and downstream LTR sequences of the viral genome (Adachi et al., 1986). These differences significantly decrease potential recombination events between the two LTRs and the excision of the provirus, which has been previously reported to potentially limit the use of the system (Battivelli et al., 2018). Two fluorescent proteins were selected for optimal sensitivity in primary cells, namely, E2-Crimson, which is transcribed from the HIV-1 LTR and serves as a marker of HIV-1 gene expression, and ZsGreen, which is transcribed from an internal promoter and serves as a marker for HIV-1 entry and integration into the host genome. The two fluorescent proteins are bright and exhibit favorable and well-separated excitation/emission profiles that allow measurement of the fluorescence intensity by separate lasers without the need for compensation (Matz et al., 1999; Strack et al., 2009). Compared to the currently used HIV_GKO_ vector (in-house constructed HIV_GKO-II_), which contains GFP and mKO2 fluorescent proteins to report HIV-1 replication and latency, HI.fate detected a higher level of replicating cells and exhibited a diminished loss of internal promoter-mediated transcription (Figure S1). We also did not detect any recombination events during the construction and modifications of HI.fate.

To analyze the fate of HIV-1 infection in different cells, we selected the EF1a-HTLV chimeric promoter as an internal promoter into our reporter vector (HI.fate.E) because of inherent high transcription activity of the promoter in different cell types. We cloned the EF1a-HTLV chimeric promoter in our vector, prepared viruses pseudotyped with the vesicular stomatitis virus (VSV) G envelope protein, and infected several T cells, including primary CD4+ T cells, as well as Cf2Th, canine thymic epithelial cells, and THP-1 (Figure 1). Infected cells were analyzed by two-color flow cytometry at 48 h (cell lines) or 96 h (primary CD4 T cells) post-infection to measure the frequency of latent cells versus cells that support HIV-1 gene expression. We observed significant variation in the fate of HIV-1 infection in the different target cells. To analyze this variability on a comparable scale, we titered the viruses on each type of cell and plotted the fluorescence profile of each cell type to assess the distribution of the latent and replicating phenotypes over an extended range of infection (we use HIV-1-mediated gene expression as a marker for replication even though HIV-1 is not fully replicating in these cells) (Figure 1C). Notably, SupT1 and Jurkat cells supported high levels of HIV-1 latency, whereas in other cells, such as H9, Cf2Th, and activated primary CD4+ T cells, active HIV-1 replication was reproducibly more dominant. Apparently, the different observed outcomes are a consequence of the unique cellular environment of each cell type. At a high multiplicity of infection, the frequency of cells that support HIV-1 replication was increased. This observation is expected because active HIV-1 LTR-mediated transcription has a dominant phenotype in our system (i.e., cells infected with two viruses, namely, one latent and the second actively expressing the E2-Crimson, are phenotypically identified as supporting HIV-1 replication because they express the E2-Crimson protein). HIV-1 entry mediated by either VSV-G-or HIV-1_KB9_ Env resulted in a similar pattern of HIV-1 fate in SupT1 cells, but VSV-G-mediated entry resulted in higher levels of viral infection (data not shown).

To optimize our reporter system and increase the sensitivity of detection of infected cells, we tested alternative internal promoters to mediate ZsGreen expression (Figure S2). We focused on strong promoters that mediate transcription of ubiquitous cellular proteins in different cell types, such as the phosphoglycerate kinase (PGK) and ubiquitin (UB) promoters, and also on strong viral promoters, such as the one of spleen focus-forming virus (SFFV). In addition, we fused ZsGreen and GFP by the self-cleavage peptide 2A to express a self-cleaved fusion protein that leads to simultaneous expression of two green fluorescence proteins. HI.fate.SFFV showed strong expression of ZsGreen and minimal interference of viral transcription from the upstream LTR promoter (Figures S2B and S2C), and it was selected for further studies. In the following sections, we show how our system can be used to study different aspects of HIV-1 fate and provide evidence that intra- and extra-cellular environments contribute to the fate of HIV-1 infection.

**Figure 2.**
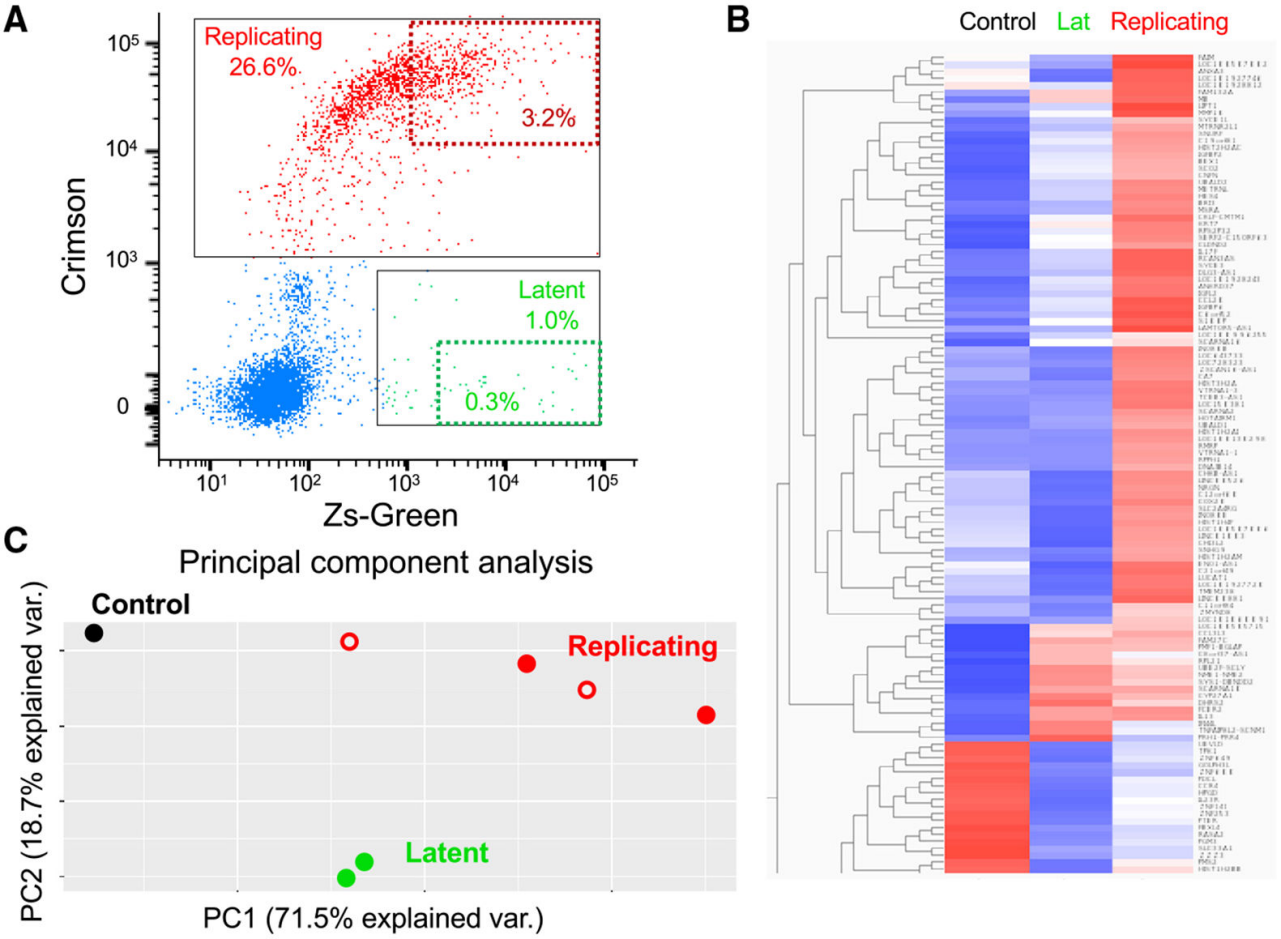
Transcriptomic analysis of HIV-1 fate in activated CD4 T cells (A) We isolated primary CD4+ T cells from a healthy individual and activated them with anti-CD3/CD28 beads prior to infection with HI.fate. SFFV viruses. We sorted the cells according to their fate but gated on cells with the most prominent phenotype (i.e., 0.3% of highest ZsGreen intensity for latent cells and 3.2% of highest ZsGreen and Crimson intensities for replicating cells; both selections are schematically shown as dashed boxes). (B) We analyzed each sorted cell population by RNA-seq and compared their gene expression. (C) Principal-component analysis evaluates differences between cells supporting different fates. Red unfilled circles represent Crimson^+^Zs-Green^−^ cells that were analyzed to detect changes related to the loss of Zs-Green expression (Figure S3). Dots were manually resized for visualization. Results represent data of two replicates for each cell population from PBMCs of a single donor, except for control uninfected PBMCs that were analyzed once.

To study the effect of the cellular environment on HIV-1 fate at high resolution, we analyzed the transcriptome of primary CD4+ T cells after infection of these cells with HI.fate viruses (Figure 2). We detected the following three different cell populations by flow cytometry: (1) cells supporting HIV-1 replication (Crimson^+^Zs-Green^+^), (2) latent cells (Crimson^−^Zs-Green^+^), and (3) exposed cells (Crimson^−^Zs-Green^−^).

**Figure 3.**
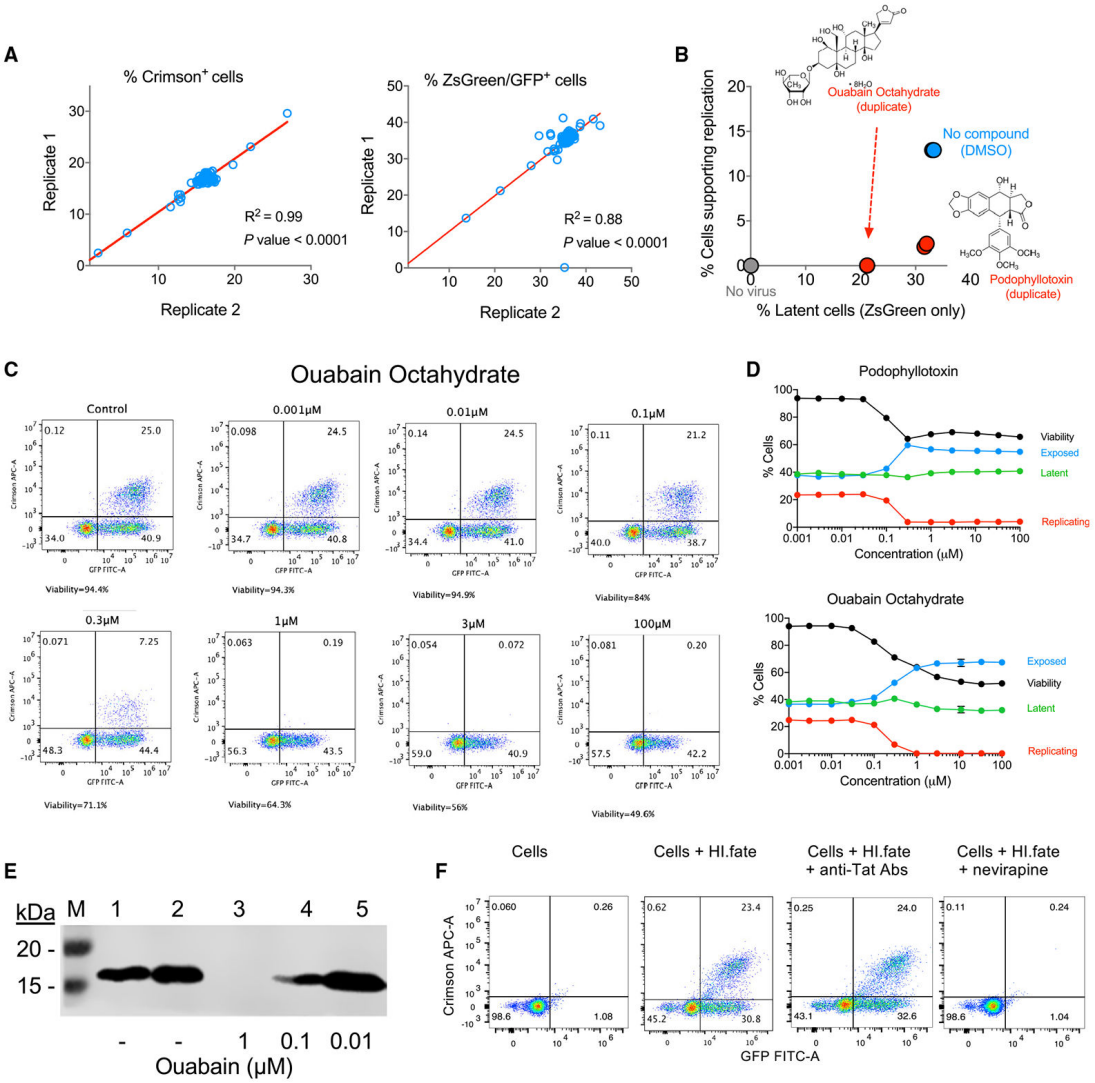
Small-molecule modulation of HIV-1 fate We incubated infected SupT1 cells with compounds from the LOPAC library and analyzed their effects on HIV-1 fate by 2-color flow cytometry. (A) Reproducibility of HIV-1 fate measurements by using HI.fate was assessed by comparing the readout from duplicate wells for each of the fluorescent proteins. (B) We analyzed compound effects by plotting the percentage of latent cells versus percentage of cells supporting replication for each compound and comparing these measurements (red) to the no-compound (light blue) and no-virus (gray) controls. The assay controls and two hit compounds are shown. (C) We retested the positive hits at different doses by using compounds that were purchased from a new batch to confirm their effects. The effects of ouabain octahydrate are shown. (D) Effects of hit compounds on 4 parameters are plotted at increasing compound concentrations (mean ± SD of percentage of cells exhibiting a specified phenotype in a represenative experiment). Exposed, Crimson^−^Zs-Green^−^ cells that are shown in the lower left quadrants of the flow cytometry analysis in (C). (E) Western blot analysis of Tat expression in 293T cells in the absence or presence of different ouabain concentrations. (F) Effects of monoclonal anti-Tat antibodies (a mixture of 1:20 dilution of 5A5.3, and 10 μg/ml of each 15.1 and 2D1.1) and non-nucleoside reverse transcriptase inhibitor (10 μM nevirapine) on HIV-1 fate. Flow cytometric results represent one of two independent experiments, with each performed in duplicate, except for initial screening and the effects of nevirapine that were done once in duplicate. Western blot analysis represents one of two independent experiments.

We sorted the infected CD4+ T cells according to HIV-1 fate and analyzed the gene expression in each population. We detected a differential expression of specific genes among three cell populations: cells supporting HIV-1 replication, latent cells, and uninfected cells. Of note, we did observe cells supporting HIV-1 replication in which Zs-Green expression was significantly reduced (Crimson^+^Zs-Green^−^; red unfilled circles in Figure 3C), but there was no significant difference in gene expression between these cells and Crimson^+^Zs-Green^+^ cells. A similar phenotype (Crimson^+^Zs-Green^−^) has been identified in other studies as well, and it was detected in only a small minority of cells in our system (Figure S1; Calvanese et al., 2013; Chavez et al., 2015). This observation suggests that in Crimson^+^Zs-Green^−^ cells, the SFFV promoter is not accessible for cellular transcription factors or that the Zs-Green transcription is subject to transcription interference from the upstream 5′-LTR promoter. We did observe that the SFFV is a weak promoter relative to the EF1a/HTLV chimeric promoter, but SFFV-mediated Zs-Green transcription did not have significant effect on the upstream transcription from HIV-1 LTR, which may generate false latent cells when using vectors containing the internal EF1a/HTLV chimeric promoter (Figure S2B). An RNA sequencing (RNA-seq) analysis of the different cell populations was robust, with approximately 4 million unique reads for every cell population (Figure S3A). A sample-sample correlation showed a reproducible analysis of duplicate samples (Figure S3D), and a principal-component analysis further confirmed the differences among the cell populations (Figures 2 and S3C). We identified 181 genes that were >2-fold downregulated and 70 genes that were >2-fold upregulated in cells that support replication compared to latent cells, and this difference was statistically significant (adjusted *P* value for multiple comparisons (*P*adj) < 0.05; Figure S4). The number of differentially expressed genes was increased when comparing cells that support HIV-1 replication and uninfected cells, and it was decreased when comparing latent and uninfected cells. All differences were statistically significant (*P*adj < 0.05; Figure S4). HDAC7 and STAT5A were among the top genes that were downregulated in cells supporting replication compared to latent cells, whereas UBALD2 and METRNAL were significantly upregulated in the comparison of the two cell populations (Figures S4 and S5; Table S1). Since the use of the two-color reporter system is relatively new, there are not yet other transcriptomic analyses of infected cells using similar experimental systems and conditions for comparison. However, we further compared our differentially expressed genes in activated CD4 T cells to a list of genes that overlapped in 2 independent transcriptome analyses of HIV-1 latency models in resting T cells (Table S2). We identified several common genes that were differentially expressed in reported latency models and in latent cells infected with HI.fate viruses (White et al., 2016). For example, the Apobec3H protein transcript and NEAT1 long non-coding RNA were both upregulated in reported analyses and in our latent cells. A prominent advantage of our system is the ability to follow the levels of gene transcripts in 3 different HIV-1 states under identical environment conditions, namely, during viral replication, during viral latency, and in uninfected cells. Such an analysis shows that expression of Apobec3H is further increased in cells supporting HIV-1 replication but that NEAT1 transcription reaches the highest levels in latent cells compared to other primary CD4 T cell populations (cells supporting replication and uninfected cells; Table S2). Our analysis also confirms a previously reported association of IFI44 with HIV-1 latency and provides additional information about the maintenance of high IFI44 expression in cells supporting HIV-1 replication (Power et al., 2015). These results provide biological insights and suggest that at least part of the determinants of HIV-1 fate in primary CD4+ T cells involves the different transcriptome in target cells.

We next studied the effects of small molecules on HIV-1 fate in SupT1 cells. We screened a small subset of 80 compounds from the library of pharmacologically active compounds (LOPAC), which is available from Millipore-Sigma. The LOPAC collection contains approved drugs, candidate drugs developed to different stages, and compounds with well-characterized activities. The LOPAC library is commonly used to validate drug discovery assays. We infected Sup T1 cells with HI-Fate viruses, followed by incubating them with 10 μM of compounds from the LOPAC collection for 48 h. We then analyzed the fate of HIV-1 by flow cytometry. Our assay was highly reproducible for the measurements of both the Crimson-positive and Zs-Green/GFP-positive cells (Figure 3A). A notable advantage of our system is the ability to simultaneously detect multiple parameters, including Zs-Green and Crimson emission, as well as side and forward scatter. Thus, we can follow the effect of any molecule in a controlled manner; for example, compounds that selectively block HIV-1 replication will decrease the number of cells that express both Crimson and Zs-Green/GFP but simultaneously increase the number of cells expressing only Zs-Green/GFP. Measurements of side and forward scatter allowed us to also monitor cell viability. Unexpectedly, our screen revealed two compounds that decreased the percentage of cells supporting replication with no significant effect on the percentage of latent cells (Figure 3B). Because chemical library compounds are typically stored for a long time, we purchased a new batch of these two compounds and tested their effects at different doses (Figures 3C and 3D). Plotting the percentage of positive cells in each population against the different compound concentrations tested suggested that the two compounds are selectively eliminating cells that support HIV-1 replication without significant effects on the other cell types. The compounds eliminated these cells at or below 1 μM, and as a result, increased the percentage of cells that were exposed; podophyllotoxin had no significant additional effects in the range of 1–100, μM and ouabain octahydrate exhibited a similar pattern with subtle differences in cytotoxicity compared to podophyllotoxin at this range of concentrations. To study the mode of action of ouabain, which was reported to decrease HIV-1 Tat secretion (Agostini et al., 2017), we tested Tat expression in our system and in 293T cells.

The level of Tat expression in T cells was below our limit of detection by using serval antibodies (data not shown). However, ouabain significantly reduced Tat expression in 293T cells in a dose-response manner that correlated with the effect of ouabain on cells supporting HIV-1 replication (Figures 3D and 3E). In addition, the infection of cells with HI.fate viruses in the presence of a mixture of anti-Tat antibodies, which could bind traces of secreted Tat protein, did not significantly affect HIV-1 fate in our system (Figure 3F). The non-nucleoside reverse transcriptase inhibitor nevirapine was used as a control and completely blocked the infection of target cells, leading to a similar phenotype as uninfected cells (Figure 3F).

**Figure 4.**
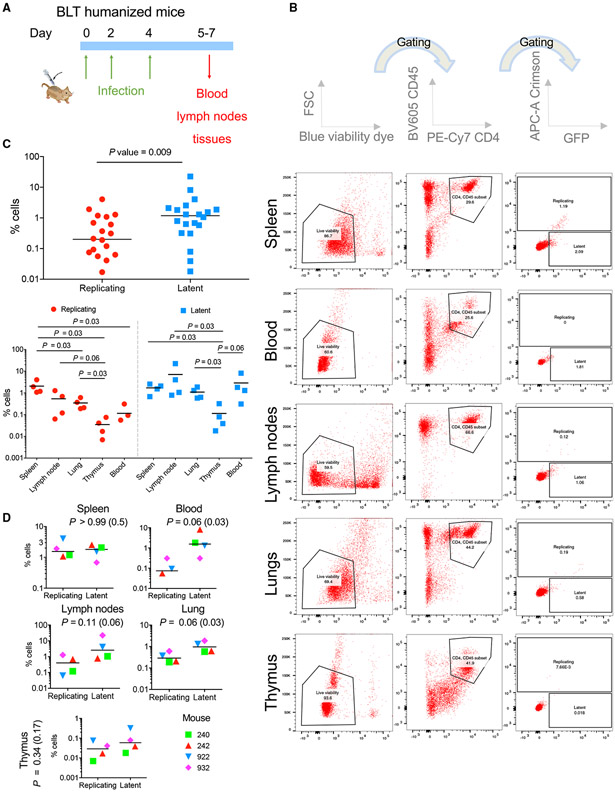
Fate of HIV-1 infection in BLT humanized model (A) Schematic layout of the experiment. (B) Flow cytometric analysis of GFP and Crimson expression in live, CD4+ and CD45+ cells from different organs of a single BLT humanized mouse. Cell populations were identified and gated, and identical gates were applied to all tissues and mice. (C) Statistical analysis of the differences between the overall number of latent cells and those supporting HIV-1 replication (top panel) and of the differences between the number of cells with specific phenotype in different organs (bottom panel) of four BLT humanized mice. (D) Statistical analysis of the differences between the number of latent cells and those supporting HIV-1 replication for each organ. For (C) and (D), the p value is two-tailed from a Mann-Whitney test, and when used, a value in parenthesis is a one-tailed p value. Results represent a single experiment with 4 BLT humanized mice.

We next studied HIV fate *in vivo* in a humanized BLT (bone-marrow-liver-thymus) mouse model. We infected 4 humanized mice intravenously with the HI.fate.SFFV virus pseudotyped with the VSV-G envelope protein every other day for 7 days to ensure efficient infection (Figure 4). The 4 humanized mice were generated from 2 different human donors (n = 2 per donor). On day 7, we sacrificed the mice and collected different organs for flow cytometric analysis. The fate of HIV infection was evaluated by measuring the green and far-red fluorescence in live, CD4+CD45+ cells present in different tissues. When we collectively analyzed cells from all organs of all four mice, we detected a significantly (p = 0.009) higher proportion of latent cells than cells supporting HIV-1 replication in our BLT mouse model (Figure 4C). Within each of these groups (replicating versus latent), we observed statistically significant differences when comparing the specific phenotype in different organs (e.g., comparing latent cells in thymus and latent cells in blood; Figure 4C). The spleen was a major source of cells supporting replication, with a comparable number of these cells and latent cells (p > 0.99; Figure 4D). Differences between the two populations were most notable in the blood, although for as yet unknown reasons, the number of positive cells isolated from the blood was significantly lower than the number of cells collected from other tissues. Specifically, for cells isolated from the blood, the number of latent cells was significantly higher than the number of cells supporting replication, and statistical significance was observed for one-tailed analysis (one-tailed p = 0.03). We observed a similar pattern in cells isolated from the lungs, where the difference between the two populations was smaller but the variability of the results among the mice was much lower. The number of latent cells in the lymph nodes and thymus was not statistically different than the number of cells supporting HIV-1 replication in these organs (Figure 4D). Overall, these results point to possible variations in the fate of HIV-1 infection in different organs, characterized by a different extra-cellular environment. Notably, we observed enrichment of latent cells in the blood, where HI.fate viruses were directly injected, and no statistical significance between the two populations (latent and replicating) in the lymph nodes where follicular helper CD4+/CXCR5+ T cells are abundant. Thus, a potential hypothesis could relate HIV-1 fate to the specific subpopulations in each organ. This view is consistent with a complex relationship between the intra- and extra-cellular environments that determines the network of cellular molecules in the cells and contribute to their interactions with HIV-1 proteins and viral replication pathways. Our observations are consistent with and add to previous knowledge on the nature of HIV-1 replication. Previous quantification of the number of HIV-1 proviruses in the reservoir of PLWH documented higher numbers of resting CD4+ T cells containing HIV-1 provirus in peripheral blood mononuclear cells (PBMCs) than in the lymph nodes (Chun et al., 1997). As resting CD4+ T cells support no or low HIV-1 replication, an increased number of proviruses is expected to result in a dominant latency state in infected cells in the blood compared to those in the lymph nodes, as observed in our study. Moreover, high HIV-1 latency in the blood and extensive replication in the lymph nodes are well-documented during the clinically asymptomatic period of HIV-1 replication in PLWH and non-human primate models (Pantaleo et al., 1993; Saksela et al., 1993). We analyzed HIV-1 fate in four humanized BLT mice, and our study provides important insights into the parameters that contribute to the fate of HIV-1 infection.

In this study, we report an improved reporter vector to monitor the fate of HIV-1 infection *in vitro* and *in vivo*. Genetic stability, ultra-bright fluorescent proteins, reproducibility, and ease of use make this vector a valuable molecular tool for its use in the scientific community. We have distributed our vector to multiple research groups worldwide and expect our system to be instrumental for studying different aspects of HIV-1 fate and biology. Here, we demonstrated the utility of our vector in the following three distinct applications: transcriptomic analysis of infected cells, screening small-molecule effectors, and *in vivo* analysis of HIV-1 fate in a humanized mouse model. These complementary studies indicate that intra- and extra-cellular environments contribute to the fate of HIV-1 infection.

## STAR★METHODS

### RESOURCE AVAILABILITY

#### Lead contact

Further information and requests for resources and reagents should be directed to and will be fulfilled by Lead Contact, Alon Herschhorn (aherschh@umn.edu).

#### Materials availability

Our system was distributed to several research groups in USA, Canada, and Europe and is available upon request.

#### Data and code availability

- RNA-seq data have been deposited at Gene Expression Omnibus (GEO) database and are publicly available as of the date of publication. Accession numbers are listed in the Key resources table.
- This paper does not report original code.
- Any additional information required to reanalyze the data reported in this paper is available from the lead contact or Zachary T. Herbert upon request

### EXPERIMENTAL MODEL AND SUBJECT DETAILS

#### Cell Lines

293T and SupT1 cells were purchased from the ATCC; THP-1 cells were differentiated using 100nM PMA; primary CD4 T cells were isolated from peripheral blood mononuclear cells (PBMC) that were purchased from StemCell Technologies. Cf2Th-CD4/CCR5 cells were generated in the laboratory of Joseph Sodroski.

#### Cell Culture

293T cells were grown in Dulbecco’s Modified Eagle Medium (DMEM) containing 10% FBS, 100 μg/ml streptomycin and 100 units/ml penicillin. Cf2Th-CD4/CCR5 cells were grown in DMEM supplemented with 400 μg/ml G418 and 200 μg/ml hygromycin B. SupT1 cells were grown in Roswell Park Memorial Institute Medium (RPMI) containing 10% FBS, 100 μg/ml streptomycin and 100 units/ml penicillin.

#### Animal Care

Humanized mouse experiments were performed according to NIH guidelines for the housing and care of laboratory animals. Protocols were reviewed and approved by the Institutional Animal Care and Use Committee (IACUC) of Massachusetts General Hospital and the University of Minnesota.

#### Generation of humanized BLT mice and *in vivo* infection

Female BLT-NOD-scid IL2Rg^−/−^ (NSG) mice (The Jackson Laboratory) were housed in a pathogen-free facility at Massachusetts General Hospital and reconstituted with human tissue as described (Brainard et al., 2009). Briefly, sublethally irradiated mice were transplanted under the kidney capsule with 1 mm^3^ fragments of human fetal liver and thymus, and injected intravenously with purified CD34^+^ human fetal liver cells to generate humanized BLT mice. Human immune cell reconstitution was monitored by flow cytometry at week 12 and week 17 post-implantation and considered sufficient if at least 200 CD4+ T cells/μl were present in peripheral blood. BLT mice showed no clinical signs of GvHD at any time during the experiment. Mice were infected 3 times intravenously, in a tail vein, at day 0, 2 and 4 in a total volume of 200 μL PBS, each time. Animals were euthanized 5 to 7 days after the first HIV infection and tissues were harvested for flow cytometry analysis.

### METHOD DETAILS

#### Plasmid Construction

All HI.fate vectors are based on the HIV-1 pNL4-3.HSA.R^−^E^−^ plasmid backbone, which was obtained from Nathaniel Landau through the NIH AIDS Reagent Program, Division of AIDS, NIAID, NIH (Cat# 3417). To construct HI.fate.E, three DNA fragments containing the 1) *e2-crimson* gene, 2) EF1a-HTLVI promoter, and 3) *zs-green* gene, were amplified by PCR. Sequences required for Gibson Assembly were added to the fragments during amplification and the three DNA fragments were cloned into the pNL4-3.HSA. R^−^E^−^ plasmid, which was digested with Not I and Xho I restriction enzymes (NEB), using Gibson Assembly (NEbuilder; NEB). All subsequent HI.fate vectors containing different internal promoters were constructed by digesting HI.fate.E with BsiW I and Sac II restriction enzymes (NEB) and cloning each promoter, which was synthesized as a gene block (IDT), into the digested vector using Gibson Assembly (NEbuilder; NEB). HI.fate vectors expressing the Zs-Green protein fused to EGFP through the 2A self-cleavage peptide were constructed by digesting HI.fate.E with BsiW I and Xho I restriction enzymes (NEB) and cloning two fragments: 1) zs-green gene, and 2) 2A-egfp gene, which were amplified by PCR, into the digested vector using Gibson Assembly (NEbuilder; NEB). HIV_GKO-II_ was constructed in-house by replacing the mCherry gene in the DuoFluoI plasmid (Calvanese et al., 2013) with the monomeric Kusabira-Orange 2 fluorescent (mKO2) gene. DuoFluoI plasmid, obtained from the NIH AIDS reagent program, was linearized using XhoI and PacI restriction enzymes. mKO2 gene was cloned into the linearized DuoFluoI plasmid along with DNA fragments containing the EF1-alpha promoter and the 3′ LTR region using Gibson Assembly according to the manufacturer’s instructions. Correct DNA sequence of the resulting HIV_GKO-II_ vector was verified by Sanger sequencing.

#### Production of Recombinant HI.fate viruses

We produced viruses as we previously described (Cervera et al., 2021; Harris et al., 2020; Herschhorn et al., 2010, 2016, 2017; Ratnapriya et al., 2020b) with the exception that we transfected 293T cells with two plasmids instead of the three plasmids routinely used for pseudovirus preparation. HI.fate (different versions) and a plasmid expressing VSV-G or HIV-1_KB9_ envelope glycoproteins were cotransfected at the mass ratio of 9:1 (9 Hi.fate / 1 Env) using Effectene (QIAGEN) according to the manufacturer’s instructions (Herschhorn et al., 2014). After a 48-hour incubation, the cell supernatant was collected and centrifuged for 5 minutes at 600-900x g at 4°C. The amount of p24 in the supernatant was measured using the HIV-1 p24 antigen capture assay (Cat# 5421, Advanced BioScience Laboratories) and the virus-containing supernatant was frozen in single-use aliquots at −80°C.

#### Viral Infection Assay

A single-round infection assay was performed in 24-well or 6-well plates by spinoculation or by adding the specified amount of p24 (or increasing amounts for virus titration) to the cells. The cells were incubated for 48 or 72 hours and analyzed by flow cytometry (Herschhorn et al., 2010). To detect the effects of anti-Tat antibodies on HIV-1 fate, a mixture of anti-tat antibodies containing 1:20 dilution of NT3 5A5.1, and 10μg/ml of each 15.1 and 2D1.1 monoclonal antibodies were added to SupT1 cells infected with HI.fate viruses, followed by incubation for 48h and flow cytometry analysis.

#### RNA-seq

Primary CD4 T cells were isolated from PBMC (1.5 E7 cells, Cat#200-0077, lot# 1608180134 from StemCell Technologies. Cells were obtained using Institutional Review Board (IRB)-approved consent forms and protocols.) as follows. Cells were thawed quickly by partially submerging the vials in a 37°C water bath. Once thawed, cells were immediately transferred to a 50 mL tube and washed with PBS + 2% FBS. After centrifuging at 300 g, 8 min and 10°C, cells were resuspended in ~100 μL medium left. Hundred μL DNase per 1 mL of cells was added and cells were incubated for 15 minutes at room temperature to prevent clumping. Cells were counted and diluted to 5E7 cells/ml with PBS + 2% FBS + 1mM EDTA. CD4 T cells were isolated using the STEMCell technologies kit for CD4 isolation according to the manufacturer’s instructions. Cells were washed and anti CD3/CD28 antibodies (1bead/cell) + IL2 50 u/ml (Milety Biotech) were added. Primary cells were activated with anti CD3-CD28 beads (Invitrogen) for 4 days and then washed, diluted to 1E6/ml and 100 μL was added to each well of 96-well plate. Cells in each well were infected with HI.fate.SFFV, which was pseudotyped with HIV-1_KB9_ envelope glycoproteins, (20ng p24/100,000 cells in 100 μl) for 2-h at 1200 g and 37°C. Cells were sorted 4.5 days post infection at Beth Israel Deaconess Medical Center core facility to 3 populations: Crimson^−^Zs-Green^+^ (~11,000 cells), Crimson^+^Zs-Green^−^ (~50,000 cells; these cells were not statistically different than Crimson^+^Zs-Green^+^ cells according to differential gene expression) and Crimson^+^Zs-Green^+^ (~110,000 cells). Uninfected primary CD4 T cells were sorted in a separate tube (~110,000 cells). Sorting took about 40 min and immediately after cells were lysed and Total RNA/DNA was isolated using QIAGEN All RNA/DNA kit. The RNA was used for cDNA prep using the SMART-seq V4 kit (Clontech) in the PCR clean station. Technical duplicates were prepared using 10 PCR cycles (processing of uninfected primary CD4 T cells resulted in no product in one replicate) and products were purified with AgenCourt AMPure beads and analyzed by Agilent bioanalyzer (DFCI MBCF). Illumina sequencing libraries were prepared from 0.5ng of cDNA and reducing Nextera XT (Illumina) reaction volumes to one half the manufacturer’s protocol to accommodate lower input amount. The finished libraries were quantified by Qubit Fluorometer (ThermoFisher Scientific), Agilent TapeStation 2200, and RT-qPCR using the Roche Kapa library quantification kit (cat # KK4854). Uniquely indexed libraries were pooled in equimolar ratios and sequenced with single-end 75bp reads on an Illumina MiniSeq (DFCI MBCF). Sequenced reads were aligned to the UCSC hg19 reference genome assembly and gene counts were quantified using STAR (v2.5.1b) (Dobin et al., 2013). Differential gene expression testing was performed by DESeq2 (v1.10.1) (Love et al., 2014) and normalized read counts (FPKM) were calculated using cufflinks (v2.2.1) (Trapnell et al., 2010). RNaseq analysis was performed using the VIPER snakemake pipeline (Cornwell et al., 2018).

#### Western Blot

293T cells were seeded in 6-well plate (5 × 10^5^ cells/well) 24h prior to transfection. Cells were transiently transfected with HIV-1 Tat-expression plasmid using effectene reagent (QIAGEN) and incubated at 37°C with 5% CO_2_ for 24h. The next day, Ouabain added at final concentrations of 1 μM, 0.1 μM, and 0.01 μM to respective wells and incubated for another 24h at 37°C with 5%CO_2_. After incubation, cell culture supernatant collected and concentrated using centrifugal concentrators (Vivaspin 6; 3kDa cut-off; Sartorius Stedim Biotech GmbH). Cells were lysed in 1X RIPA lysis buffer (150mM NaCl, 50mM Tris pH 8, 1.0% IGEPAL, 0.5% Sodium deoxycholate, 0.1% SDS) supplemented with 1mM PMSF protease inhibitor, incubated at 4°C for 15-20min, centrifuged at 12000xg for 5 min and the supernatant was collected. Samples were separated by SDS-PAGE and the proteins were transferred to a nitrocellulose membrane. We detected the HIV-1 tat protein using 1:200 dilution of anti-tat NT3 5A5.1 monoclonal antibody followed by antimouse (1:10,000 dilution) conjugated to horseradish peroxidase.

SupT1 cells were seeded (5x10^5^ cells/well) in 24-wells plate, infected by HI.fate viruses and incubated at 37°C with 5% CO_2_. After 48h, infected cells were transferred to T-25 flasks (5ml/flask), 25 μM and 0.1 μM Ouabain were added to separate flasks of infected cells, keeping one flask as untreated control. Cells were incubated for further 48 hours at 37°C with 5% CO_2_ and culture supernatants and lysate samples were analyzed by western blot as described above.

#### Flow cytometry

Human lymphocyte characterization was performed on an LSR II (BD Biosciences). Lungs from BLT mice were minced using scissors and incubated in medium containing Liberase (0.02 mg/ml) and DNase (5 mg/ml) (both from Sigma-Aldrich). Spleen, liver, thymus and lymph nodes were minced using scissors. Processed tissues were passed through a 40-μm mesh strainer to obtain single-cell suspensions. Remaining red blood cells were removed using RBC lysis buffer (Sigma-Aldrich). Cells were stained using the anti-human monoclonal antibodies fluorophore-conjugated (Biolegend) Brilliant Violet 605 anti-human CD45 (HI30), and PE/Cy7 anti-human CD4 Antibody (clone RPA-T4). Dead cells were excluded using LIVE/DEAD Fixable Blue Dead Cell Stain Kit (Life Technologies). Cells were fixed with 2% paraformaldehyde after staining. Whole blood (100 μL) was directly stained with antibodies and treated with FACS lysing solution (BD Biosciences) before analysis. CountBright Absolute Counting Beads (ThermoFisher) were used to determine cell numbers.

### QUANTIFICATION AND STATISTICAL ANALYSIS

We used the DESeq2 (v1.10.1) package in R for statistical analysis of RNA-seq data. For the BLT humanized mouse experiments, we used the Mann-Whitney test in GraphPad Prism 7 to determine statistical significance differences between groups and reported two-tailed *P values*. In some cases, we additionally reported one-tailed *P values* in parenthesis and indicated this analysis in the figure legend.

## Supplementary Material

1

Table S1

Table S2

Table S3

## Figures and Tables

**Table:** KEY RESOURCES TABLE

REAGENT or RESOURCE	SOURCE	IDENTIFIER
Antibodies		
Anti-HIV-1 tat Monoclonal (NT3 5A5.1)	NIH AIDS Reagent Program	Cat# ARP-4374
Anti-HIV-1 tat Monoclonal (15.1)	NIH AIDS Reagent Program	Cat# ARP-1974
Anti-HIV-1 tat Monoclonal (NT3 2D1.1)	NIH AIDS Reagent Program	Cat# ARP-4138
Anti-human IgG HRP	Jackson Immunolaboratories	Cat# 709-036-098; RRID: AB_2340497
PE/Cy7 anti-human CD4 Antibody (clone RPA-T4)	Biolegend	Cat# 300511; RRID: AB_314080
Brilliant Violet 605 anti-human CD45 (HI30)	Biolegend	Cat# 304041; RRID: AB_2562105
Chemicals, peptides, and recombinant proteins		
Effectene Transfection Reagent	QIAGEN	Cat# 301425
Podophyllotoxin	Sigma	Cat# P4405-50MG
Ouabain Octahydrate	Sigma	Cat# O3125-250MG
Nevirapine	NIH AIDS Reagent Program	Cat# 4666
Dulbecco’s Phosphate Buffered Saline (PBS)	Sigma	Cat# D8537-500ML
Fetal Bovine Serum (FBS)	GIBCO	Cat# 10437-010
Penicillin-Streptomycin (PenStrep)	GIBCO	Cat# 15140-122
Dulbecco’s Modified Eagle Medium (DMEM)	GIBCO	Cat# 11965-084
Roswell Park Memorial Institute Medium (RPMI)	GIBCO	Cat# CX30306
Triton X-100	Sigma	Cat# X100-100ML
Dimethyl Sulphoxide (DMSO)	Sigma	Cat# D2438-10ML
Hydrogen Peroxide Solution (30% w/w)	Sigma	Cat# H1009-100ML
Tween-20	BIO-RAD	Cat# 170-6531
Critical commercial assays		
HIV-1 p24 antigen capture assay	Advanced BioScience Laboratories	Cat# 5421
Deposited data		
RNA-seq (Intra- and extra-cellular environments contribute to the fate of HIV-1 infection)	This manuscript	GEO: GSE180044
RNA-seq (Primary CD4+ T cells control)	This manuscript	GEO: GSM5448986
RNA-seq (Primary CD4+ T cells, Latent_1)	This manuscript	GEO: GSM5448987
RNA-seq (Primary CD4+ T cells, Latent_2)	This manuscript	GEO: GSM5448988
RNA-seq (Primary CD4+ T cells, Replicating [low green] 1)	This manuscript	GEO: GSM5448989
RNA-seq (Primary CD4+ T cells, Replicating [low green] 2)	This manuscript	GEO: GSM5448990
RNA-seq (Primary CD4+ T cells, Replicating 1)	This manuscript	GEO: GSM5448991
RNA-seq (Primary CD4+ T cells, Replicating 2)	This manuscript	GEO: GSM5448993
Experimental models: Cell lines		
HEK293T/17 cells	ATCC	Cat # CRL-11268
Cf2Th CD4/CCR5 cells	Laboratory of Joseph Sodroski	Parental Cf2Th cells are from ATCC (CRL-1430)
Sup T1 cells	ATCC	Cat# CRL-1942
Primary CD4+ T cells	StemCell Technologies	Cat# 200-0077
Experimental models: Organisms/strains		
Female BLT-NOD-scid IL2Rg−/− (NSG) mice	Jackson Laboratory	N/A
Recombinant DNA		
HI.fate.E	This study	N/A
HI.fate.SFFV	This study	N/A
HIV-1 pNL4-3.HSA.R-E-	NIH AIDS Reagent Program	Cat# 3417
DuoFluo	NIH AIDS Reagent Program	Cat# ARP-12595
HIV-1_KB9_ Env	Laboratory of Alon Herschhorn / Joseph Sodroski	N/A
VSV-G	Laboratory of Alon Herschhorn / Joseph Sodroski	N/A
Software and algorithms		
FlowJo	FlowJo, LLC (BD)	https://www.flowjo.com/solutions/flowjo/downloads

## Highlights

- HI.fate reports HIV-1 fate in target cells by using sensitive fluorescent proteins
- Alternative fates of HIV-1 infection are linked to differential gene expression
- Small molecules can modulate the fate of HIV-1 infection
- Extra-cellular environment contributes to the fate of HIV-1 *in vivo*
