# Supplementary material for: Intra- and extra-cellular environments contribute to the fate of HIV-1 infection
Source: Cell Rep. Author manuscript; Available in PMC 2021 Sep 24. (PMC8463096; doi:10.1016/j.celrep.2021.109622)
Supplement: 1 [file NIHMS1737333-supplement-1.pdf]

Cell Reports, Volume *36*

Supplemental information

Intra- and extra-cellular environments contribute to the fate of HIV-1 infection

Sneha Ratnapriya, Miranda Harris, Angela Chov, Zachary T. Herbert, Vladimir Vrbanac, Maud Deruaz, Vasudevan Achuthan, Alan N. Engelman, Joseph Sodroski, and Alon Herschhorn

Supplemental Information

Intra- and extra-cellular environments contribute to the fate of HIV-1 infection

Sneha Ratnapriya, Miranda Harris, Angela Chov, Zachary T. Herbert, Vladimir Vrbanac, Maud Deruaz, Vasudevan Achuthan, Alan N. Engelman, Joseph Sodroski, and Alon Herschhorn

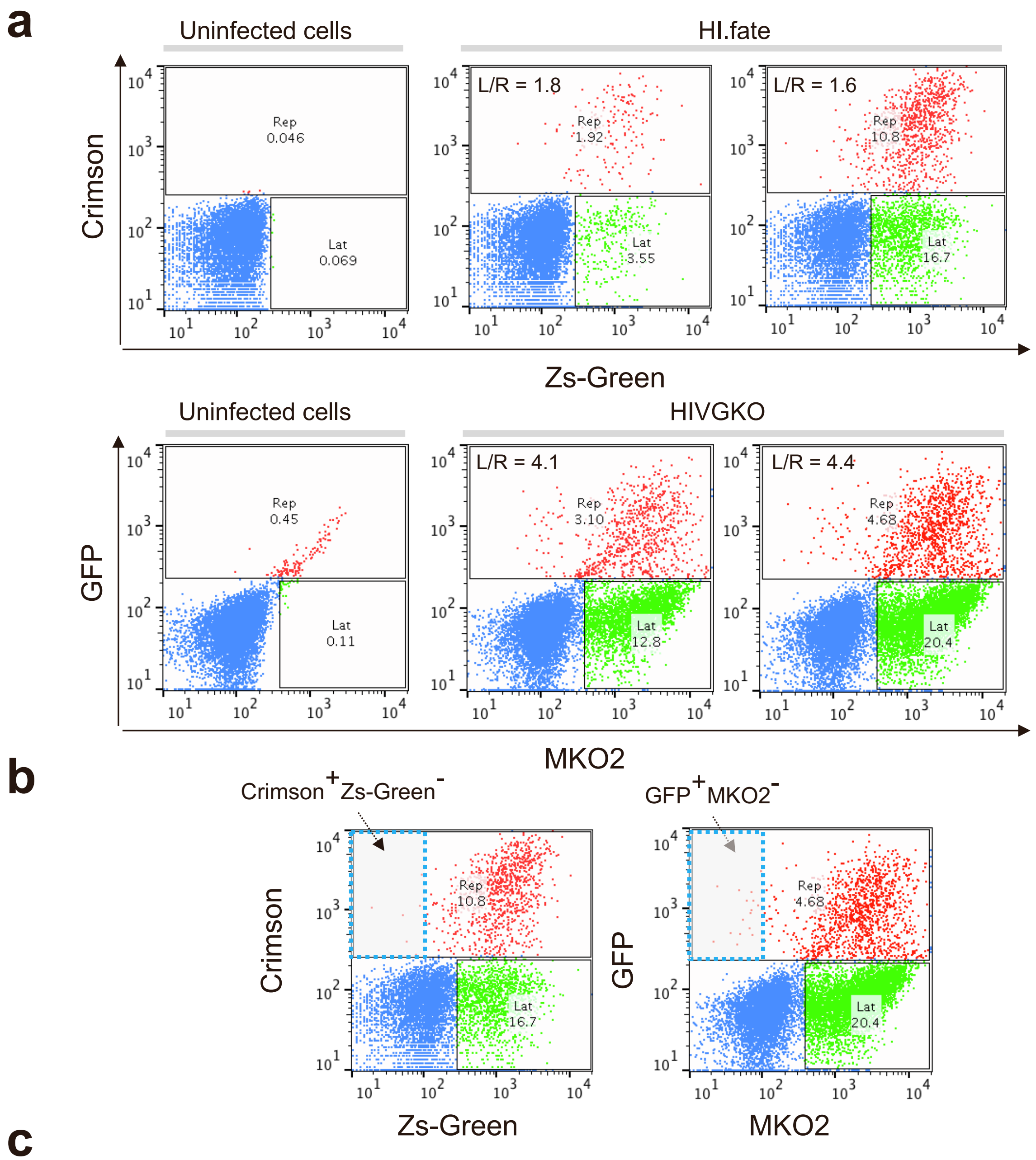

c

```
HI.fate     TGGAAGGGCTAATTCACTCCCAAAGAAGACAAGATATCCTTGATCTGTGGATCTACCACACACAAGGCTACTTCCCTGAT 80
HIVGKO-II   -----------------------C--------------------------------------------------------
HI.fate     TGGCAGAACTACACACCAGGGCCAGGGGTCAGATATCCACTGACCTTTGGATGGTGCTACAAGCTAGTACCAGTTGAGCC 160
HIVGKO-II   ---------------------------A---------------------------------------------------A
HI.fate     AGATAAGGTAGAAGAGGCCAATAAAGGAGAGAACACCAGCTTGTTACACCCTGTGAGCCTGCATGGAATGGATGACCCTG 240
HIVGKO-II   ---G-----------A------G--------------C----------------------------G-----------G-
HI.fate     AGAGAGAAGTGTTAGAGTGGAGGTTTGACAGCCGCCTAGCATTTCATCACGTGGCCCGAGAGCTGCATCCGGAGTACTTC 320
HIVGKO-II   ----------A---------------------------------------A-----------------------------
HI.fate     AAGAACTGCTGACATCGAGCTTGCTACAAGGGACTTTCCGCTGGGGACTTTCCAGGGAGGCGTGGCCTGGGCGGGACTGG 400
HIVGKO-II   ----------------------------------------------------------------------*---------
HI.fate     GGAGTGGCGAGCCCTCAGATGCTGCATATAAGCAGCTGCTTTTTGCCTGTACTGG 455
HIVGKO-II   -----------------------------------------------T--------
```

Figure S1. Comparison of HI.fate and HIV_{GKO-II} vectors. Related to Figure 1. (**a**) PVs prepared using HI.fate and HIV_{GKO-II} vectors were used to infect Jurkat cells and infected cells were analyzed 48 hours post infections for expression of the related fluorescent proteins by flow cytometry. L/R denotes the ratio of latent cells to those supporting viral replication (%Lat/%Rep).(**b**) Cells that support HIV-1 replication but lost internal promoter-mediated transcription are shown in the gray area for total infection of 27.5% (HI.fate) and 25.1% (HIV_{GKO-II}) of Jurkat cells by the related viruses. (**c**) Alignment of the DNA sequence of the U3 region (HIV-1 promoter) of the two vectors. Differences are shown in red in the HI.fate sequence. "-", identical nucleotides, "*", deletion.

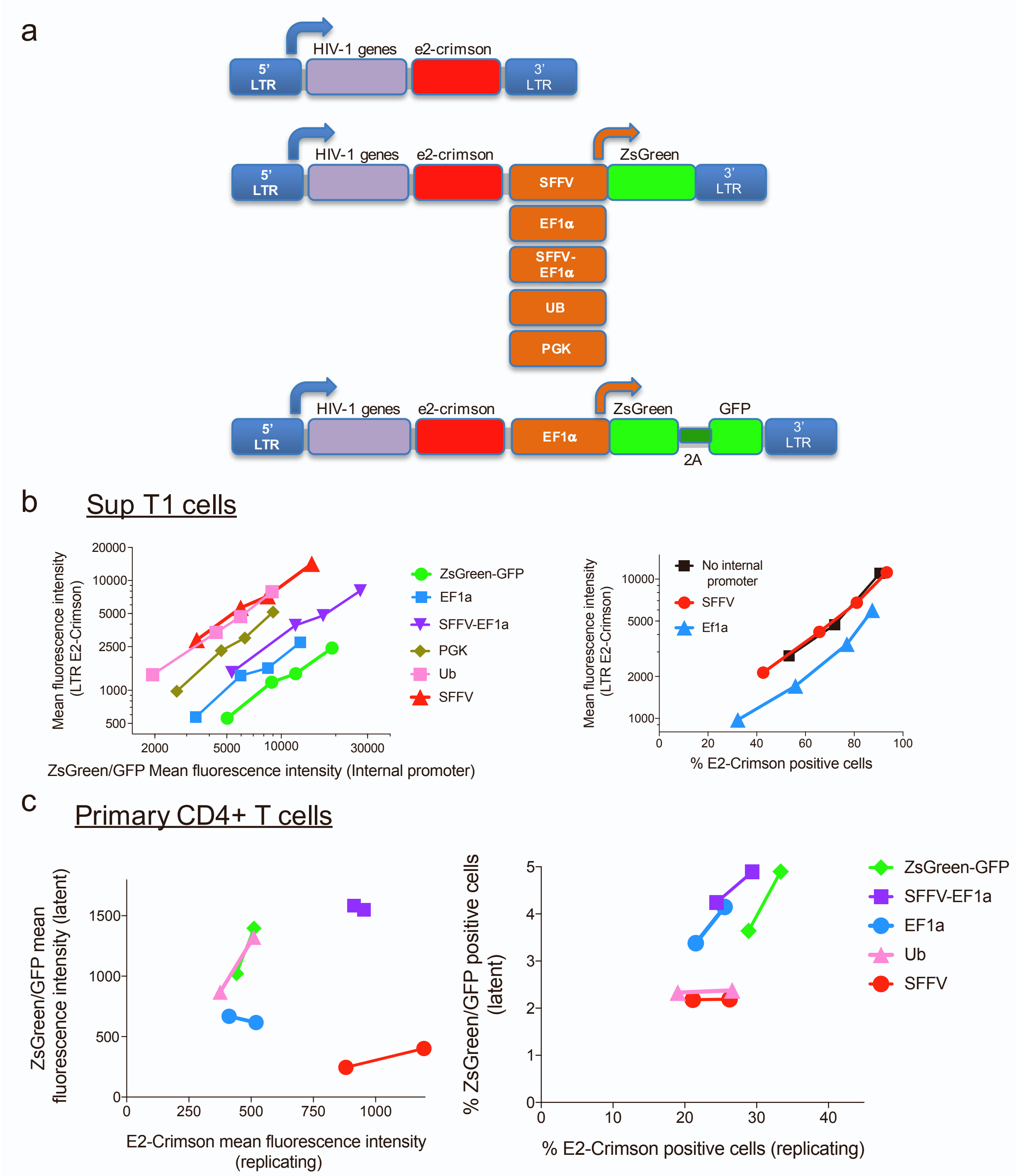

Figure S2. The effects of internal promoters and fused green fluorescent proteins on HIV-1 transcription. Related to Figure 1. (**a**) A schematic representation of the different vectors that were tested in the study. HI.fate was engineered to include the specified internal promoters or the ZsGreen fused to GFP via the 2A self-cleavage peptide for simultaneous expression of two green fluorescence proteins and increased sensitivity of detection. (**b**) Sup T1 cells were infected with increasing amounts of HI.fate viruses that were prepared by using the different vectors shown in **a**. Left – mean fluorescence intensity (MFI) of LTR-mediated expression versus MFI of internal promoter-mediated expression for different internal promoters. Right – MFI and frequency of cells supporting HIV-1 gene expression. Note that HI.fate.SFFV exhibit similar E2-Crimson as a vector with no internal promoter, indicating that the SFFV promoter does not significantly interfere with the upstream LTR-mediated transcription. (**c**) Primary CD4 T cells were isolated from PBMC by negative selection and activated with anti CD3/CD28 beads prior to infection. Cells were infected with 20ng or 100ng p24 of the indicated HI.fate viruses. Left – relationship between the MFI of latent cells and cells supporting HIV-1 gene expression for different internal promoters; Right – similar to the left panel but the % positive cells were plotted.

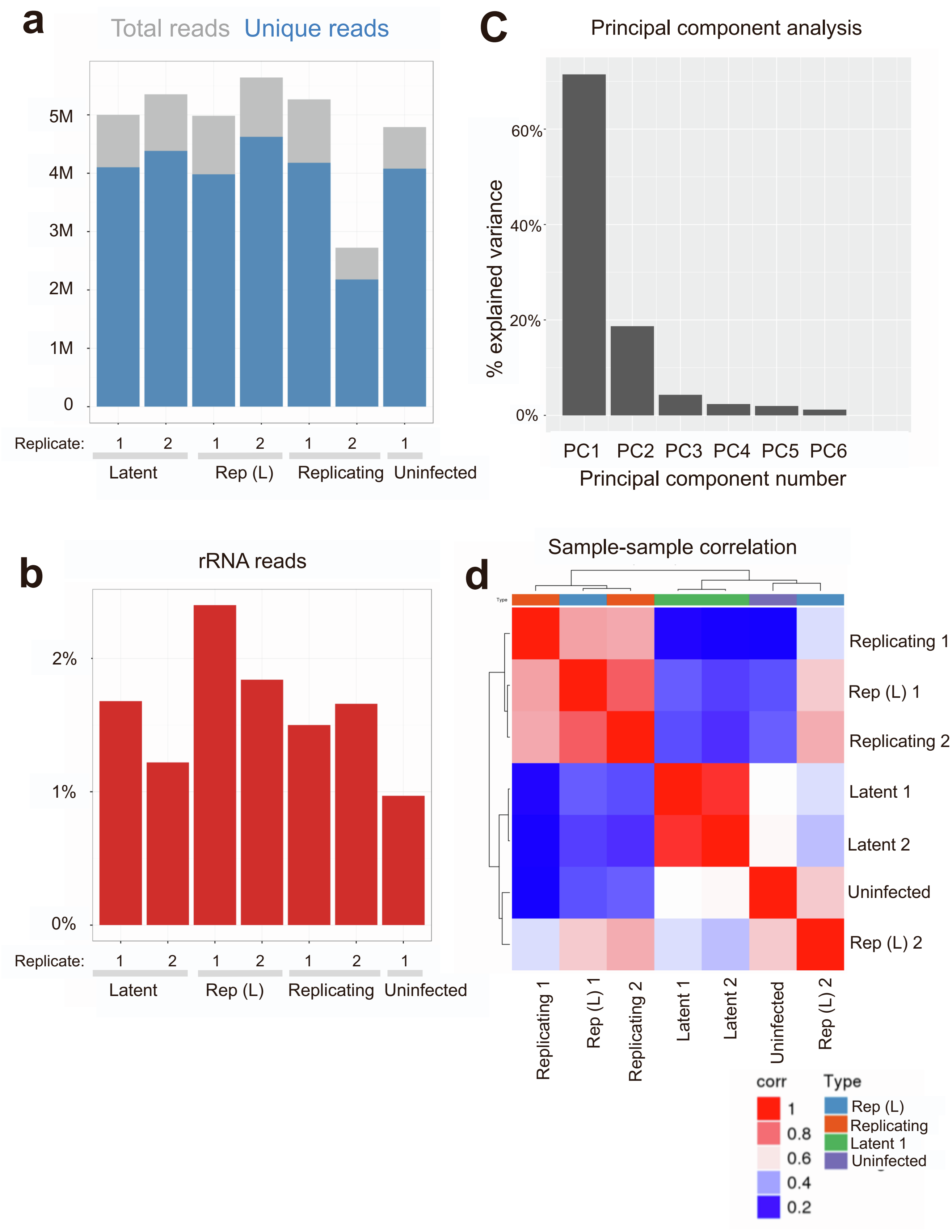

Figure S3. VIPER - Visualization Pipeline for RNAseq.[30] Related to Figure 2. (**a**) Total and unique reads during RNA-seq analysis of each of the specified samples. (**b**) The fraction of rRNA among the RNA-seq reads. (**c**) Extended principal component analysis of the variance between the four groups. (**d**) Correlation among the different groups/samples. Rep (L), Crimson^{+}ZsGreen^{-} cell population.

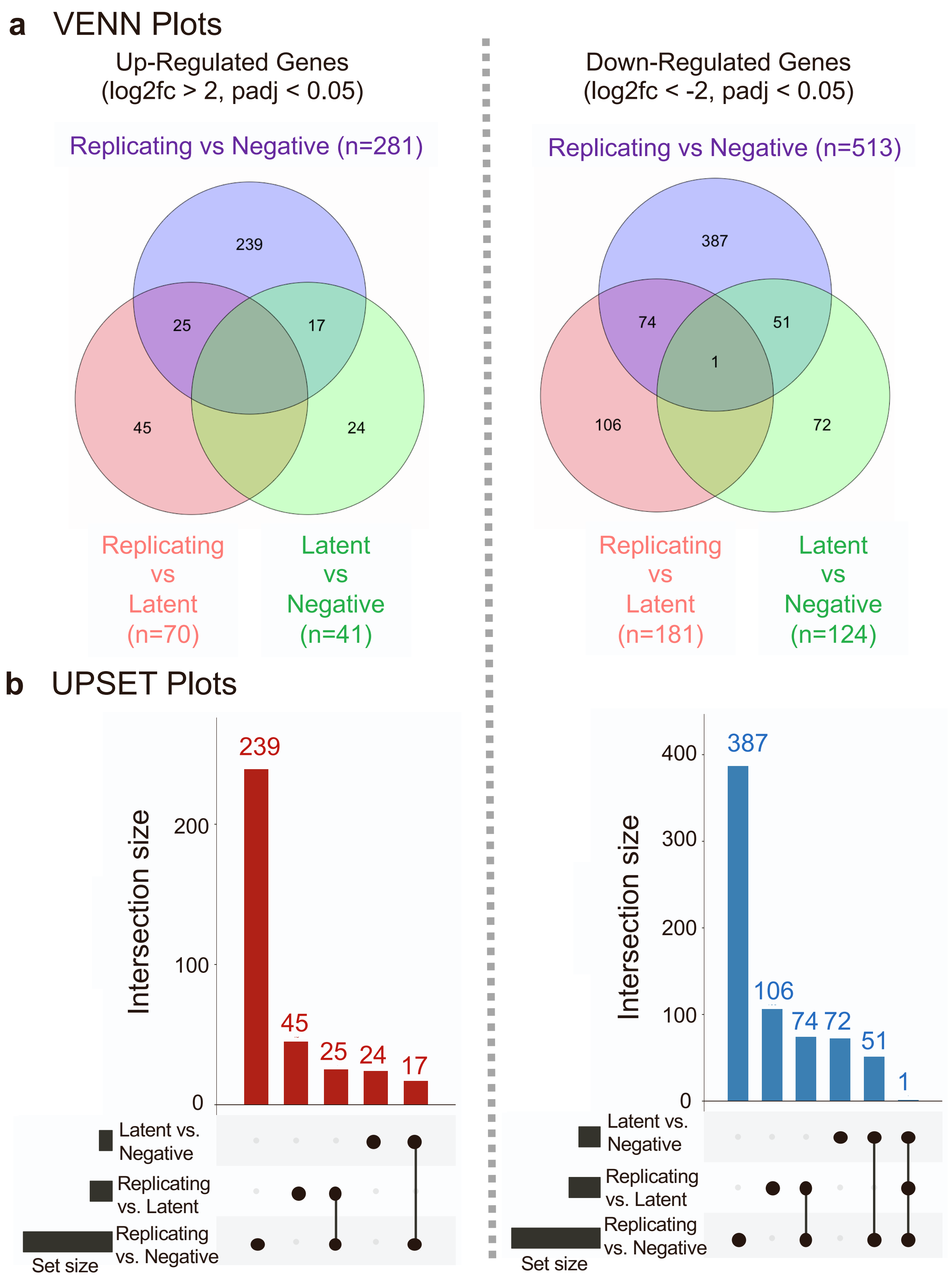

Figure S4. Differential gene analysis. Related to Figure 2. (**a**) VENN and (**b**) UPSET plots showing the number of unique and overlapping gene from DESeq2 results with adjusted p-value < 0.05 and foldchange >2 for the upregulated genes and < -2 for the downregulated genes for each of the comparisons; Replicating versus Negative, Replicating versus Latent, and Latent versus Negative. P value was adjusted (Padj) for multiple comparisons.

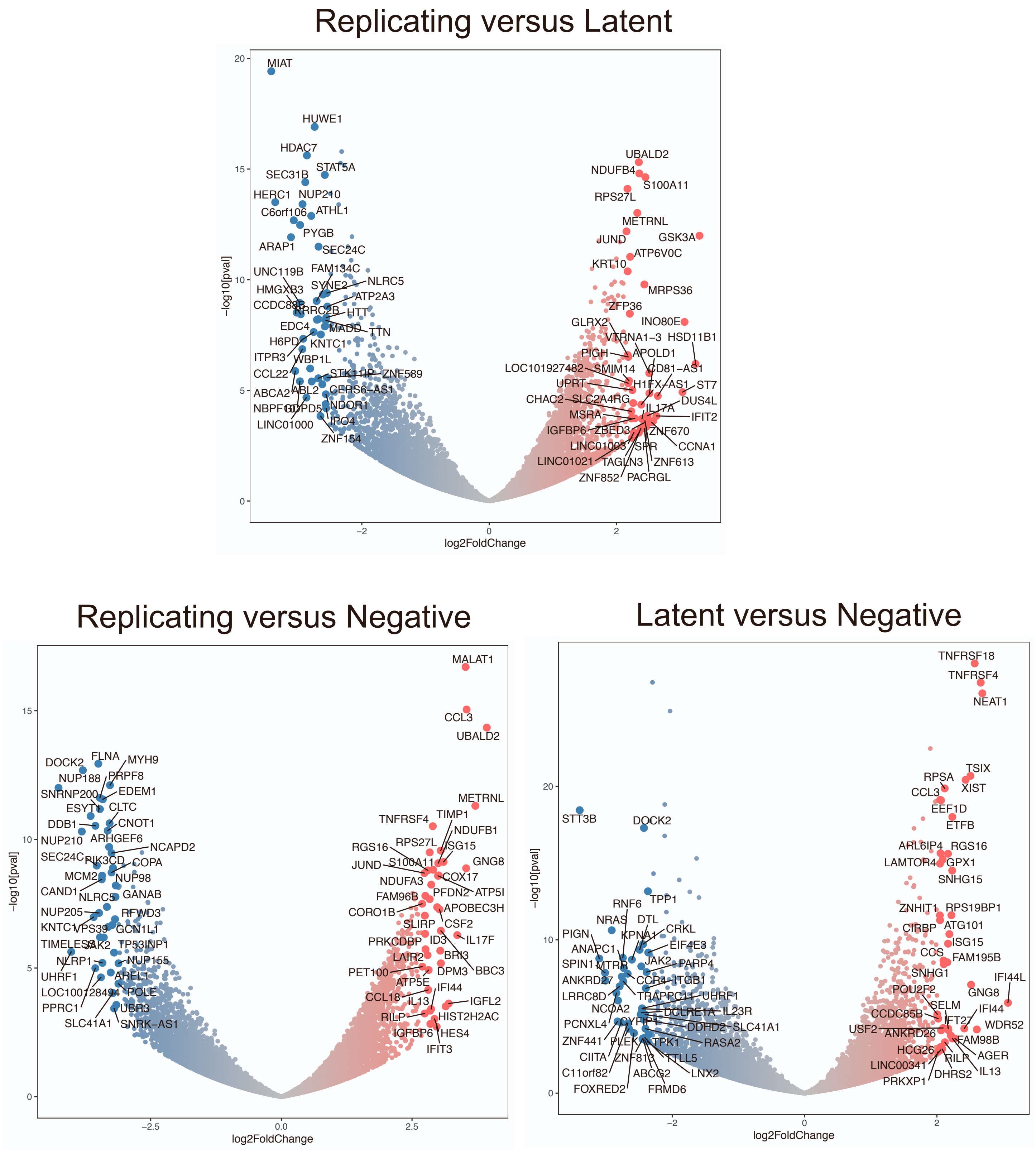

Figure S5. Volcano plots for the differential gene expression between the specified groups. Related to Figure 2. The plots showing log2 fold change and p-value from DESeq2 results. Upregulated genes are shown in red and downregulated genes are shown in blue. The top 40 upregulated and downregulated genes based on foldchange are labeled with the gene symbol. Plots were generated by VIPER - Visualization Pipeline for RNAseq (Cornwell et al., 2018).
